# Sex Differences in the Association of Simultaneous Decline in Blood Pressure and Decline in Cognition during Aging

**DOI:** 10.1002/ana.78280

**Published:** 2026-07-07

**Authors:** Nicholas W. Baumgartner, Ana W. Capuano, Lisa L. Barnes, David A. Bennett, Zoe Arvanitakis

**Affiliations:** ^1^ Rush Alzheimer's Disease Center Rush University Medical Center Chicago IL USA; ^2^ Department of Neurology University of Chicago Chicago IL USA

## Abstract

**Objective:**

Both high and declining blood pressure (BP) are associated with risk of cognitive decline in older adults, and women have higher rates of hypertension and faster cognitive decline in late‐life. In old age, the relationship between changes in BP and changes in cognition is complex, and although sex differences in cognitive function and decline are recognized, little is known about the relationship of longitudinally collected BP and cognition, simultaneous changes in BP and cognition, or whether they differ by sex. We investigated the association of simultaneous change in BP and cognition in older adults followed annually for an average of 9 years.

**Methods:**

A total of 4,719 older adults without baseline dementia (mean age = 76.7 [standard deviation = 7.7] years; 74% women) from 5 prospective community‐based cohort studies completed annual assessments of BP and cognition, yielding composite global and 5 domain‐specific scores. BP and cognition were modeled simultaneously using joint bivariate linear mixed‐effects models.

**Results:**

Among 3,502 women and 1,217 men, faster decline in systolic BP was associated with faster decline in global cognition (*r* = 0.20, *p* < 0.01). Stratified models revealed a stronger relationship in women (*r* = 0.26, 95% confidence interval [CI]: 0.17–0.37) than men (men: *r* = 0.01, 95% CI: −0.13 to 0.11). In secondary analyses, decline in systolic BP was related to decline in 5 cognitive domains in women but none in men.

**Interpretation:**

This large longitudinal study reports evidence of a relationship between simultaneous decline in systolic BP and cognition in women, but not men. As women experience a disproportionate burden of both hypertension and cognitive decline in late‐life, declining systolic BP, a routine clinical measure, may serve as an important indicator of concurrent cognitive decline in older women. Understanding these sex‐specific associations may help advance the understanding of drivers behind sex differences in cognitive decline. ANN NEUROL 2026;100:572–583

High blood pressure (BP) and cognitive impairment are common in older adults.^1^ Numerous cross‐sectional and longitudinal studies indicate that hypertension in midlife (ages 40–64 years) and late‐life (ages 65+) is associated with increased risk of cognitive decline.^2^, ^3^ However, associations between BP and cognition are more complex and less understood in old age, with accumulating evidence suggesting low BP in late‐life is also associated with cognitive impairment.^2^ Systolic BP (SBP) and diastolic BP (DBP) increase from childhood through adulthood, but data support a decrease in BP late in life.^4^, ^5^, ^6^ Although the exact age at which SBP begins to decline is unclear, some studies suggest an inverted‐U relationship with SBP reaching its peak around the seventh decade.^6^, ^7^ Declines in SBP have been associated with increased dementia risk,^8^, ^9^ poorer Mini‐Mental State Exam (MMSE) performance,^10^, ^11^ higher mortality,^12^, ^13^ and a greater number of infarcts in the postmortem brain.^14^ However, decline in BP has been observed before the onset of cognitive decline, particularly among very old adults,^11^, ^15^ and it remains unclear whether declining BP represents a preclinical manifestation of neuropathology or contributes causally to cognitive decline.

To date, sex differences in the relationship between BP and cognition remain poorly characterized. Although men exhibit higher rates of hypertension in early and midlife, women have a greater prevalence after age 65^16^ and comprise nearly two‐thirds of all persons affected by Alzheimer's disease,^17^ suggesting a disproportionate burden on women later in life. Several sex‐specific factors may contribute, including hormonal changes related to menopause, and that women compared to men have lower rates of antihypertensive treatment use and lower educational attainment, all of which have been associated with increased risk of hypertension and cognitive decline.^18^, ^19^, ^20^ Although sex differences in cognitive function and decline are established,^21^ relatively few studies have investigated sex differences in the relationship between BP and cognition.^10^, ^22^, ^23^ Most longitudinal studies have relied on baseline BP to predict cognition years later, limiting insight into how changes in BP and cognition unfold simultaneously. Moreover, the limited number of studies that have collected repeated assessments of BP and cognition have relied on brief screening tools (ie, MMSE), which are less sensitive, suffer from ceiling effects, and do not capture domain specific deficits. Understanding these relationships could enhance precision medicine, identifying individual risks associated with cognitive decline to help reduce disparities by sex, improve patient outcomes, and guide better clinical decision‐making.

Using data from more than 4,700 older adults across 5 ongoing longitudinal, community‐based cohort studies of aging, we investigated sex differences in the relationship between long‐term changes in BP and cognitive decline in late‐life. Building on the limited existing literature, we conducted novel analyses examining simultaneous change in BP and change in global and domain‐specific cognitive function, to elucidate the complex relationship between BP and cognitive aging by sex. Based on prior associations between decline in BP and risk of dementia^8^, ^9^ and studies showing changes in BP and cognition disproportionately affect older women,^16^, ^17^ we hypothesized that faster declines in BP would be related to faster declines in cognitive function, with stronger relationships in women compared to men.

## Methods

### Study Design and Population

Participants were drawn from 5 ongoing longitudinal, prospective, community‐based cohort studies of aging conducted at the Rush Alzheimer's Disease Center: the Religious Orders Study (ROS),^24^ the Rush Memory and Aging Project (MAP),^25^ the Minority Aging Research Study (MARS),^26^ the Rush Alzheimer's Disease Center Clinical Core (ClinCore),^27^ and the Latino Core (LATC).^28^ These studies share similar design and standardized data collection, enabling the combination of harmonized data across the 5 cohorts. All studies were approved by the Rush University Medical Center institutional review board and performed in accordance with the Declaration of Helsinki. Authors reviewed and adhered to the Strengthening the Reporting of Observational Studies in Epidemiology (STROBE) checklist for cohort studies.

ROS, initiated in 1994, enrolls Catholic clergy from approximately 40 convents and monasteries across the United States. MAP, initiated in 1997, enrolls older community members from retirement and subsidized housing facilities in the Chicago area. MARS (initiated in 2004) and ClinCore (initiated in 2008) enroll older Black adults and LATC (initiated in 2015) enrolls older Latino/Hispanic adults, primarily from churches, senior housing, and social clubs in Chicago and the surrounding suburbs. All participants enrolled without known dementia and provided written informed consent for annual clinical evaluation. Follow‐up rates range from 85 to 90%.^24^, ^25^, ^26^, ^27^

Details regarding study design and other study features have been published previously.^24^, ^25^, ^26^, ^27^, ^28^ Briefly, baseline and annual evaluations included in‐home physical and neurological evaluation, neuropsychological testing, and a detailed medical history with direct visual inspection of all medications (including antihypertensives) and over‐the‐counter agents (eg, vitamins/supplements) taken in the prior 2 weeks. Annual follow‐up visits were essentially identical to the baseline evaluation. Self‐reported demographics included age (from date of birth), sex (male/female), years of education, race/ethnicity, and medical history (eg, history of hypertension, age at menopause). Other data from the history, physical examination (eg, BP [see below], height and weight to derive body mass index [BMI], etc.), and blood draws (eg, apolipoprotein E genotyping [*APOE ε4*]) were available, as previously published.^29^, ^30^, ^31^

### Participants

Participants were eligible if they had complete baseline data for the main covariates of interest and at least 2 measurements (baseline and at least 1 follow‐up) of SBP, DBP, and composite global cognitive function. Of 5,394 enrolled participants, 465 had not yet completed a follow‐up visit (required to model change) and 208 were excluded because of missing data (79 missing baseline BP or cognitive data, and 129 had fewer than 2 measures of either BP or global cognition because of missing data). To ensure comparable ages across sexes, participants younger than 50 or older than 100 at baseline were excluded (2 persons removed, both men), yielding a final analytic sample of 4,719 participants. Compared to those included, excluded individuals (n = 675 [of which 509 were women]) were older (*t* = 3.73, *p* < 0.01), had lower educational attainment (*t* = −5.15, *p* < 0.01), worse global (*t* = −9.73, *p* < 0.01) and domain specific cognitive performance (−7.26 > *t*‐values >−8.55, *p*‐values <0.01), higher prevalence of hypertension (χ^2^ = 5.44, *p* = 0.02), shorter follow‐up duration (χ^2^ = 1350.84, *p* < 0.01), and higher mortality during follow‐up (χ^2^ = 35.72, *p* < 0.01). Sex differences observed in the analytic sample were largely replicated among excluded participants, with only 2 exceptions. Excluded men were older than excluded women (*p* < 0.01), whereas no age difference was observed among included participants, and no difference in BMI was observed among excluded individuals (*p* = 0.38), although women had slightly higher BMI among included individuals.

### Procedures

BP was assessed annually using an automated sphygmomanometer. Three readings (2 seated and 1 standing) were taken 1 minute apart and averaged to calculate mean SBP and DBP as previously reported.^32^ SBP and DBP were standardized into z‐scores using the baseline mean and standard deviation (SD) of the entire cohort. Clinical evaluations documented demographics, medical history, medications, and BMI.

Cognitive function was assessed annually using a comprehensive battery of 19 neuropsychological tests covering multiple cognitive domains, as detailed previously.^33^ The tests were organized into composites, capturing global cognition and 5 specific cognitive domains, with higher scores indicating better cognitive performance. Briefly, 3 working memory (Digit Span forward and backward; Digit Ordering), 3 semantic memory (Boston Naming; Verbal Fluency; Reading Test), 7 episodic memory (Word List Memory, Recall and Recognition; immediate and delayed recall of the East Boston Story; immediate and delayed recall of Story A of the Wechsler Memory Scale‐Revised), 4 perceptual speed (Symbol Digit Modalities Test; Number Comparison; 2 indices from a modified version of the Stroop Test), and 2 visuospatial abilities (Line Orientation; Progressive Matrices) tests were administered. Raw test scores were standardized into z‐scores using the baseline mean and SD of the entire cohort. Composite scores for each domain were calculated by averaging the z‐scores for all tests within that domain. The global cognitive function composite score was derived by averaging z‐scores across all 19 tests.

### Statistical Analysis

Baseline characteristics were summarized using means and SDs or frequencies (Table 1), with sex differences assessed using *t*‐tests or χ^2^ tests as appropriate. Linear mixed‐effects models were used to estimate changes in SBP, DBP, global cognition, and 5 cognitive domains over time. All models were adjusted for age (centered at 76.7 years), education (centered at 15.8 years), antihypertensive medication use (1 = yes, 0 = no), sex (when applicable) and interactions of these variables with time.

**TABLE 1:** Analytic Sample Characteristics

	Total (n = 4,719)	Women (n = 3,502)	Men (n = 1,217)
Demographic			
Age (yr)	76.7 ± 7.7	76.7 ± 7.8	76.8 ± 7.5
Race/ethnicity (n, %)			
White, non‐Latino	3,057, 64.8	2,201, 62.9	856, 70.3
Black, non‐Latino	1,243, 26.3	976, 27.9	267, 22.0
Latino	387, 8.2	305, 8.7	82, 6.7
Other	32, 0.7	20, 0.6	12, 1.0
Education (yr)	15.8 ± 3.9	15.5 ± 3.8	16.5 ± 4.3
Annual follow‐up visits (yr)	8.7 ± 5.7	8.8 ± 5.6	8.2 ± 5.8
Clinical			
Systolic blood pressure (mmHg)	134.9 ± 19.0	135.2 ± 19.3	134.0 ± 18.3
Diastolic blood pressure (mmHg)	76.2 ± 11.5	76.4 ± 11.6	75.8 ± 11.4
Systolic blood pressure slope (mmHg/yr)	−0.02 ± 0.04	−0.02 ± 0.04	−0.02 ± 0.03
Diastolic blood pressure slope (mmHg/yr)	−0.02 ± 0.03	−0.02 ± 0.03	−0.02 ± 0.04
History of hypertension^a^ (n, %)	2,644, 56.0	2034, 58.1	610, 50.1
Antihypertensive medication use (n, %)	3,142, 66.6	2,322, 66.3	820, 67.4
Body mass index (kg/m^2^)	28.2 ± 5.5	28.3 ± 5.9	27.93 ± 4.3
Age at menopause (yr)	46.8 ± 7.0	46.8 ± 7.0	n/a
Died during study (n, %)	2,591, 54.91	1814, 51.80	777, 63.85
Cognition			
Global cognitive function score	0.03 ± 0.62	0.06 ± 0.60	−0.04 ± 0.66
Working memory	0.03 ± 0.78	0.03 ± 0.78	0.02 ± 0.79
Semantic memory	0.04 ± 0.81	0.06 ± 0.81	−0.02 ± 0.81
Episodic memory	0.04 ± 0.74	0.09 ± 0.72	−0.12 ± 0.79
Perceptual speed	0.05 ± 0.83	0.11 ± 0.81	−0.10 ± 0.86
Visuospatial ability	0.03 ± 0.82	−0.04 ± 0.79	0.23 ± 0.85

Values are mean ± standard deviation unless otherwise noted; all variables represent value at first baseline visit unless otherwise specified.

^a^ Self‐reported prior diagnosis.

Primary analyses used joint bivariate mixed‐effects regression analyses, outcomes were both BP (SBP and DBP modeled separately) and global cognition modeled simultaneously, used to determine whether change in BP was related to change in cognition. The models were then repeated stratified by sex. These models estimated simultaneously the levels and rates of change in BP and cognition, leveraging multiple years of data for more accurate and robust slope estimates. The association (ie, covariance) of these levels and rates of change were characterized by a joint distribution of the 4 random effects (ie, the covariance between the 2 intercepts and 2 slopes). From the derived covariance–covariance G‐matrix (see Table S1), the correlation between level and the correlation between rate of change were calculated for all outcomes. To assess sex differences, 95% confidence intervals (CIs) for the correlation between rates of change ascertained using the bivariate mixed effect model were calculated through Fisher transformations. We, then, applied a conservative bootstrapping analysis (50 runs; mean, 30 hours run time) to confirm significant differences in the correlation, reported here. Secondary analyses repeated the fully adjusted simultaneous bivariate mixed‐effects regression analyses in the full sample and stratified by sex, replacing global cognition with individual cognitive domains 1 at a time.

A series of sensitivity analyses were conducted to assess whether other covariates or sources of bias (ie, reverse causality, survival bias, among others) could explain the observed associations. In all repeated simultaneous bivariate mixed‐effects models, each additional covariate was entered individually along with its interaction with time. Given prior evidence that obesity,^34^, ^35^, ^36^ race (Black compared to White),^37^, ^38^ and *APOE ε4* status^39^, ^40^, ^41^ influence the relationship between hypertension and cognition, models were repeated adding BMI, race/ethnicity (non‐Latino White, non‐Latino Black, Latino, and other), and *APOE ε4* status (no *ε4* allele = 0, presence of any *ε4* allele = 1) as covariates. To address potential differences across cohorts and follow‐up duration, models were repeated adding study cohort (ROS, MAP, MARS, ClinCore, or LATC) and number of follow‐up visits as covariates. Reverse causality (ie, cognitive decline driving BP decline) was assessed by excluding participants who developed dementia within 3 years of baseline. Survival bias (given shorter life expectancy for men^42^) was assessed by adding a covariate for death (alive = 0, died = 1) during follow‐up. To examine whether the effect may differ by age category, all models were repeated stratified by baseline age (≤70 vs >70 years old).

To explore potential explanations for the observed sex‐differences, *t*‐tests and χ^2^ tests were conducted to determine which covariates known to influence cognition (diabetes status, high‐density lipoprotein (HDL) ratio, lipid medication, physical activity, smoking history, and vascular risk score) differed by sex in our sample. Variables that differed by sex were added individually, and models were repeated. Finally, given women that undergo menopause at earlier ages are at increased risk for hypertension^43^ and experience earlier cognitive decline,^44^ we added age at menopause as an additional covariate in models testing associations in women only.

All analyses were conducted using SAS/STAT software, version 9.4 (SAS Institute, Cary, NC), with a 2‐sided α = 0.05.

## Results

**FIGURE 1:**
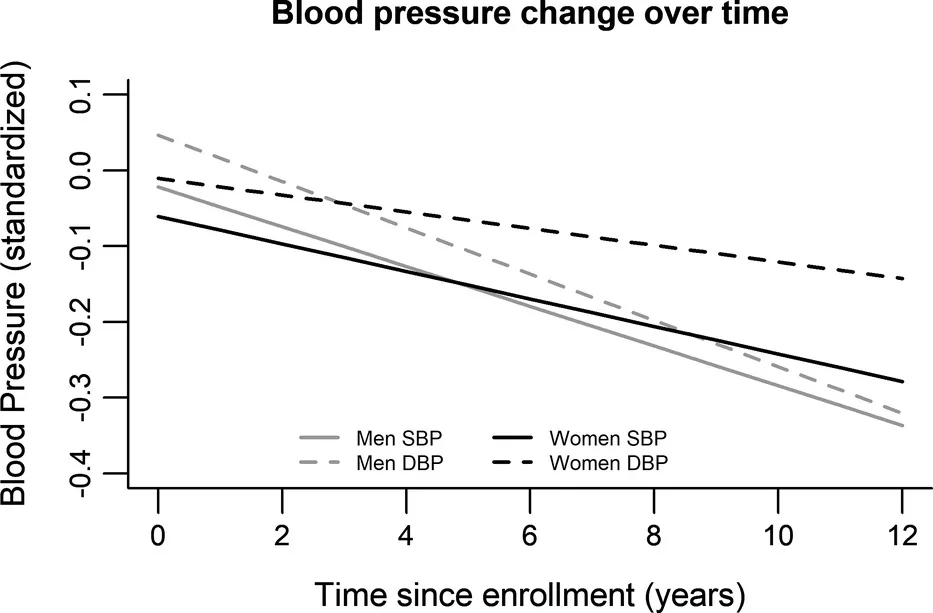
Longitudinal trajectories of systolic blood pressure (SBP) (solid line) and diastolic blood pressure (DBP) (dashed line) separately for women (black) and men (gray) derived from fully adjusted linear mixed effect models. Both SBP (z‐score) and DBP (z‐score) decreased significantly over time (*p* < 0.01). Although rate of SBP decline did not differ by sex, men exhibited a faster rate of DBP decline than women (*p* < 0.01). Covariates included age, sex, education, antihypertensive use, and their interactions with time.

Baseline characteristics for the full sample and stratified by sex are summarized in Table 1. Average number of follow‐up visits was 8.7 ± 5.7 years (mean ± SD, range = 1–29.6 years). In adjusted linear mixed‐effect models (Table 2) SBP, DBP, and all cognitive measures declined over time in the full sample and separately by sex (*p*‐values <0.01). Men exhibited a faster decline in DBP than women (*p* < 0.01) (Fig 1), but no other sex differences in rates of decline were observed.

**TABLE 2:** Estimates of Change in Blood Pressure and Change in Cognition

Outcomes	Total (n = 4,719)	Women (n = 3,502)	Men (n = 1,217)	Sex difference
	Estimate (± SE)	Estimate (± SE)	Estimate (± SE)	*p*
Systolic pressure	−0.019 ± 0.003**	−0.018 ± 0.003	−0.026 ± 0.005	0.07
Diastolic pressure	−0.013 ± 0.003**	**−0.011 ± 0.003**	**−0.031 ± 0.005**	**<0.001**
Global cognition	−0.091 ± 0.003**	−0.092 ± 0.003	−0.089 ± 0.005	0.62
Working memory	−0.058 ± 0.002**	−0.060 ± 0.002	−0.080 ± 0.006	0.20
Semantic memory	−0.113 ± 0.004**	−0.114 ± 0.004	−0.109 ± 0.007	0.97
Episodic memory	−0.089 ± 0.003**	−0.091 ± 0.004	−0.056 ± 0.004	0.45
Perceptual speed	−0.103 ± 0.002**	−0.104 ± 0.003	−0.097 ± 0.005	0.50
Visuospatial ability	−0.047 ± 0.002**	−0.047 ± 0.003	−0.047 ± 0.004	0.91

Results of mixed‐effects models adjusted for age, education, antihypertensive medication use, and sex (when not stratified by sex). *p*‐Value column represents the interaction between sex and time, indicating different rates of change between women and men. Significant sex differences are **bolded** for clarity.

** *p* < 0.01.

Primary results of the simultaneous bivariate mixed‐effects regression analyses investigating simultaneous change in BP and global cognitive function across the full sample revealed that higher level (intercept terms) of SBP was associated with lower level of global cognition at study entry. Similarly, the slope (time terms) of SBP was associated with the slope of global cognition, indicating faster decline in SBP was related to a faster decline in global cognition. Although higher DBP level was associated with lower global cognition, change in DBP was not related to change in global cognition (Table 3).

**TABLE 3:** Correlations of Level and Change of Blood Pressure with Level and Change of Cognition and 5 Cognitive Domains

	Level		Change	
	*r* value	*p*	*r* value	*p*
Systolic pressure				
Global cognition	**−0.04** *	**0.04**	**0.20** **	**<0.001**
Working memory	−0.02	0.31	**0.24** **	**<0.001**
Semantic memory	**−0.04** *	**0.04**	**0.21** **	**<0.001**
Episodic memory	−0.01	0.61	**0.15** **	**<0.001**
Perceptual speed	**−0.07** **	**<0.001**	**0.18** **	**<0.001**
Visuospatial ability	**−0.06** **	**<0.01**	**0.16** **	**<0.001**
Diastolic pressure				
Global cognition	**−0.06** **	**<0.01**	−0.03	0.35
Working memory	**−0.05** **	**<0.01**	−0.04	0.28
Semantic memory	**−0.04** *	**0.03**	−0.03	0.45
Episodic memory	**−0.05** *	**0.01**	−0.05	0.15
Perceptual speed	**−0.05** **	**<0.01**	−0.01	0.79
Visuospatial ability	**−0.06** **	**<0.01**	0.01	0.84

Level indicates the intercept and change indicates the slope of blood pressure and the cognitive variable. *p*‐Value reflects the covariance parameter estimate (data not shown). Significant correlations are **bolded** for clarity.

* *p* < 0.05.

** *p* < 0.01.

**FIGURE 2:**
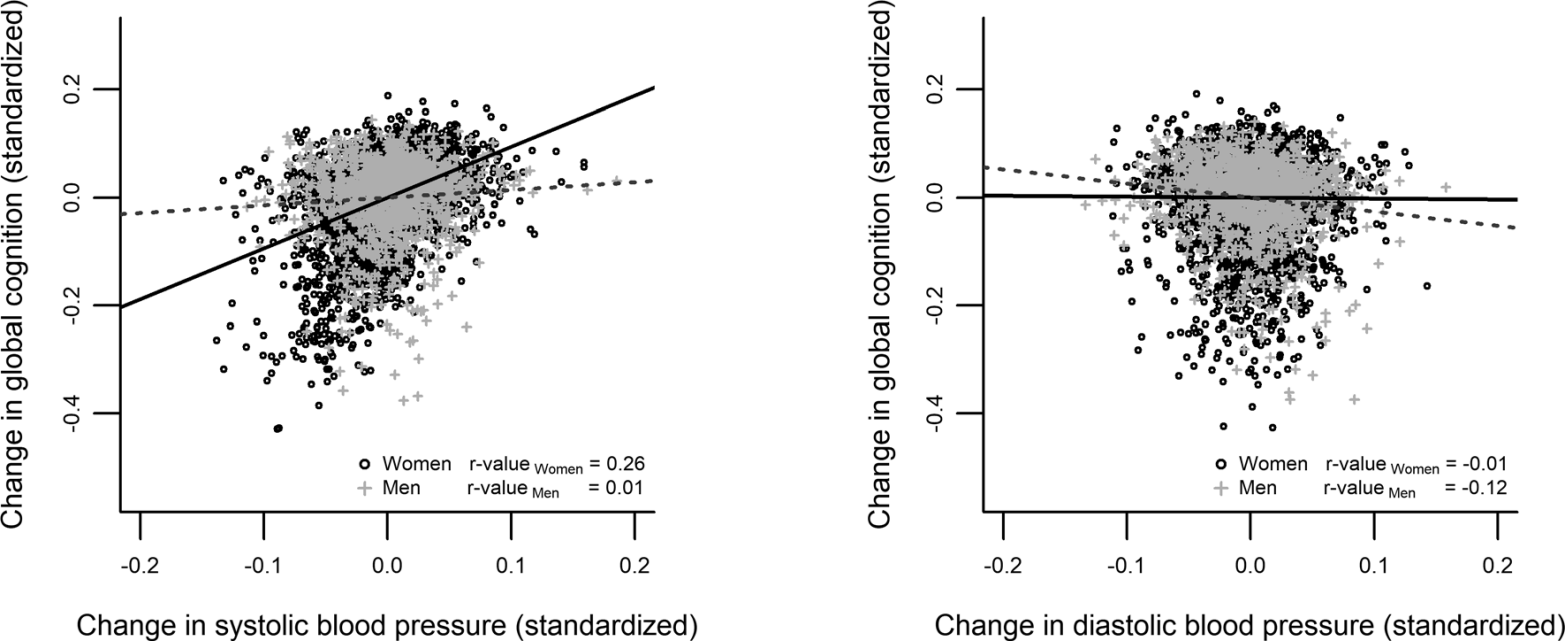
Scatterplots depicting the correlation between change in blood pressure (BP) and change in composite global cognition for women and men, derived from the variance–covariance matrix from joint bivariate mixed‐effects models. Change in BP and change in global cognition are both presented in standardized units (z‐score). Left panel: In women (black line) decline in systolic BP (SBP) was associated with decline in global cognition (*r* = 0.26, *p* < 0.01), whereas in men (gray line) there was no association (*r* = 0.01, *p* = 0.94). Right panel: Change in diastolic BP (DBP) was not associated with change in global cognition for either sex.

**FIGURE 3:**
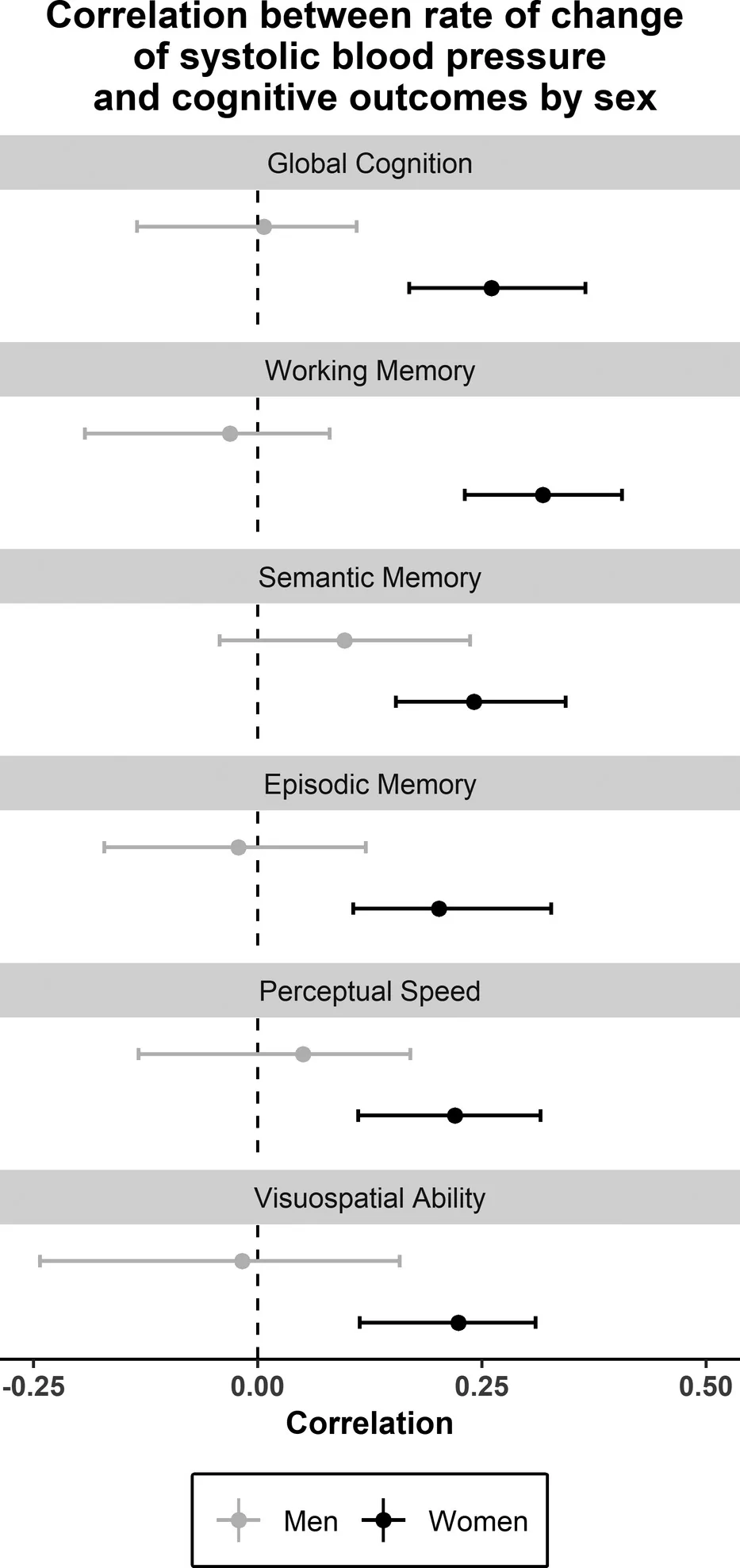
Bootstrapped 95% confidence intervals derived from simultaneous fully adjusted bivariate mixed‐effects regression models display the correlation between change in systolic blood pressure (SBP) and change in composite global cognition and across 5 separate domains. In women (black) decline in SBP was significantly correlated with decline in global cognition and across all 5 domains, while correlations for men (gray) were not statistically different from 0. Sex‐differences were observed, such that compared to men, women exhibited stronger correlations between decline in SBP and both decline in global cognition and decline in working memory.

When stratified by sex (Table 4), level of SBP in women was unrelated to level of global cognition, however, in men higher level of SBP was associated with lower global cognition score at study entry. Decline in SBP was related to decline in global cognition in women, but not in men (Fig 2). Specifically, bootstrap CIs revealed sex differences in this relationship (Fig 3), such that women exhibited a stronger correlation between decline in SBP and decline in global cognition (*r* = 0.26, 95% CI: 0.17–0.37) than men (*r* = 0.01, 95% CI: −0.13–0.11). For DBP, higher level of DBP at study entry was associated with lower level of global cognition in both sexes at study entry, but change was not related to cognition (see Fig 2).

**TABLE 4:** Correlations of Level and Change of Blood Pressure with Level and Change of Cognition and 5 Cognitive Domains by Sex

	Women				Men			
	Level		Change		Level		Change	
	*r* value	*p*	*r* value	*p*	*r* value	*p*	*r* value	*p*
Systolic pressure								
Global cognition	−0.03	0.15	**0.26** **	**<0.001**	**−0.08** *	**0.03**	0.01	0.94
Working memory	−0.02	0.49	**0.32** **	**<0.001**	−0.05	0.17	−0.03	0.69
Semantic memory	**−0.05** *	**0.04**	**0.24** **	**<0.001**	−0.04	0.29	0.10	0.23
Episodic memory	0.00	0.87	**0.20** **	**<0.001**	−0.05	0.16	−0.02	0.76
Perceptual speed	**−0.07** **	**<0.01**	**0.22** **	**<0.001**	**−0.08** *	**0.03**	0.05	0.48
Visuospatial ability	**−0.06** **	**<0.01**	**0.22** **	**<0.001**	**−0.08** *	**0.04**	−0.02	0.83
Diastolic pressure								
Global cognition	**−0.05** *	**0.01**	−0.01	0.84	**−0.11** **	**<0.01**	−0.12	0.09
Working memory	**−0.05** *	**0.03**	0.00	0.99	−0.07	0.06	**−0.18** *	**0.03** *
Semantic memory	−0.04	0.10	−0.01	0.77	−0.07	0.07	−0.08	0.30
Episodic memory	−0.03	0.11	−0.04	0.35	**−0.10** **	**<0.01**	−0.10	0.18
Perceptual speed	−0.04	0.08	0.03	0.46	**−0.11** **	**<0.01**	−0.04	0.56
Visuospatial ability	**−0.06** **	**<0.01**	0.01	0.78	−0.07	0.05	0.00	0.96

Level refers to the intercept and change refers to the slope of blood pressure and the cognitive variable, respectively. *p*‐Value reflects the covariance parameter estimate (Table S1). Significant correlations are **bolded** for clarity.

* *p* < 0.05.

** *p* < 0.01.

Secondary analyses investigating cognitive domains in the full sample showed that higher levels of SBP were inversely related to levels of semantic memory, perceptual speed, and visuospatial abilities, but not working memory or episodic memory performance at study entry (Table 3). Faster decline in SBP was related to faster declines across all 5 domains. Higher level of DBP was associated with lower levels of performance across all 5 domains at study entry, but changes in DBP were not related to changes in any cognitive domains over time.

In analyses stratified by sex (Table 4), higher level of SBP was associated with lower levels of semantic memory (women), perceptual speed (women and men), and visuospatial abilities (women and men), but not working memory or episodic memory at study entry. Women with faster decline in SBP had faster declines in all 5 cognitive domains, whereas no relationship existed for men in any domains. Bootstrap analyses revealed sex differences, such that women exhibited a stronger correlation between decline in SBP and decline in working memory (*r* = 0.32, 95% CI: 0.23–0.41) compared to men (*r* = −0.03, 95% CI: −0.19–0.08), as shown in Figure 3. For DBP, higher level at study entry was associated with lower levels of working memory (women), episodic memory (men), perceptual speed (men), and visuospatial ability (women), but not semantic memory at study entry. In men, increasing DBP was related to faster decline in working memory, but was not related to change in any domain for women.

Sensitivity analyses to address a range of potential factors demonstrated the findings were robust. Adjustment for BMI, race/ethnicity, *APOE ε4* status, study cohort, or number of follow‐up visits did not alter the relationships between change in BP and change in cognition (global or any of the 5 domain measures) in the full sample or in sex‐stratified models. Excluding participants who developed dementia within 3 years of baseline did not alter the relationships, suggesting reverse causality as a less likely explanation. Likewise, adjusting for death during follow‐up did not change the results, suggesting survival bias is unlikely to explain the findings. Additional sensitivity analyses explored potential contributors to the observed sex differences. Although the proportion with diabetes, high cholesterol (HDL ratio), physical activity levels, and smoking history differed between men and women (*p*‐values <0.001), adjusting for these factors did not attenuate the observed sex differences. Adjustment for age at menopause likewise did not attenuate the findings, suggesting these biological, metabolic, behavioral, and vascular factors are unlikely to explain the observed sex differences.

Age‐stratified models indicated that relationships between changes in BP and cognition were primarily observed later in life. Among participants older than 70 years old (n = 3,656), relationships between declining SBP and global or domain specific cognition were comparable or had even larger effect sizes relative to the primary models. In sex‐stratified analyses, the same relationships were present in older women as in the primary models, in most cases with larger effect sizes. Older men showed similar relationships between DBP and working memory (*r* = −0.19, *p* = 0.03), and a relationship with global cognition also emerged (*r* = −0.18, *p* = 0.03). Among participants 70 years old or younger at baseline (n = 1,063), relationships were attenuated and had smaller effect sizes (*r* = 0.00–0.12) relative to primary models. In sex‐stratified analyses, relationships were similarly attenuated in women under age 70, with only working memory (*r* = 0.18, *p* = 0.04) and visuospatial ability (*r* = 0.22, *p* = 0.02) remaining, but reduced in effect size. No relationships were observed in men under age 70. Results of age‐stratified sensitivity analyses in the full sample (Table S2) and stratified by sex (Table S3) are reported.

## Discussion

In this large, community‐based longitudinal study of more than 4,700 older adults examined annually for an average of 9 years, we found faster declines in SBP were associated with faster simultaneous declines in cognition, and consistent with our hypothesis, relationships were stronger in women. Faster decline in SBP was related to faster decline in global cognition and across all 5 cognitive domains in women, whereas no relationships were observed in men. A significant sex difference was identified for global cognition and working memory, indicating the relationship with declining SBP was stronger in women compared to men. Conversely a faster increase in DBP was related to a faster decline in working memory in men but not women, however, no sex difference was observed. As the medical field moves toward precision medicine, our findings suggest declining SBP, a measurement routinely monitored in clinics worldwide, may be an important correlate of concurrent cognitive decline in women.

Few longitudinal studies have assessed how change in BP relates to concurrent change in cognitive function, as most longitudinal studies have relied on baseline BP to predict future cognitive outcomes. For instance, higher baseline SBP or DBP have been shown to predict worse processing speed, visuospatial ability, immediate memory, and executive function 8 to 13 years later.^45^, ^46^, ^47^ Conversely, a limited number of studies have found that low baseline BP was associated with lower MMSE scores at follow‐up,^46^, ^48^ in line with the current results. Longitudinal studies with repeated BP and cognitive assessments have mainly shown sustained hypertension is associated with greater MMSE decline compared to normotensive individuals,^49^, ^50^ but low baseline BP has been associated with faster annual decline in MMSE scores^51^ and decline in SBP (measured at baseline and once again at study completion) was associated with annual decline in MMSE scores over the same period.^10^, ^11^ Notably, these studies with multiple measurements have mainly relied on cognitive screening tools (eg, MMSE) rather than comprehensive assessments of global and domain‐specific cognition. The present study extends this literature by reporting for the first time that the decline in SBP is related to concurrent decline in global cognition and across 5 cognitive domains using composite measures assessed annually, suggesting that declining SBP in older adults may signal broader cognitive decline across multiple domains, advocating for monitoring of BP trends as an integral part of cognitive health assessments.

We conducted pre‐specified sex‐stratified analyses to further explore sex differences in the relation of change in BP with change in cognition. In support of our hypothesis, women demonstrated stronger relationships between changes in BP and changes in cognition compared to men. Specifically, decline in SBP was related to declines in global cognition and all 5 cognitive domains for women, whereas no relationships were observed in men. Additionally, sex differences were identified, with women showing a stronger relationship between decline in SBP and declines in both global cognition and working memory compared to men. Although past studies show older women have higher rates of hypertension^16^ and increased risk of cognitive decline^21^ and Alzheimer's disease dementia,^52^ the sex‐specific relationship between changes in BP and cognition remains poorly understood, often because of limited data at follow‐up or a reliance on brief cognitive screening tools. The few studies that have investigated these relationships generally find that higher SBP is related to worsening performance across several cognitive domains in women, with no relationships in men.^22^, ^23^ One study has reported that SBP decline from baseline predicted lower MMSE scores 3 years later, only for women^10^; however, the relatively small sample of men (n = 223), reliance on the MMSE, and a short follow‐up period limit conclusions about differences by sex. Our findings extend this work, reporting decline in SBP and declines across multiple cognitive domains in women and supporting sex differences for global cognition and working memory. Regarding DBP, we observed that faster increases were related to a faster decline in working memory in men, but not women. However, to our knowledge, sex differences in the relationship between DBP and working memory have not been reported, although 1 study of men only showed lower DBP at age 50 predicted better working memory performance 20 years later,^53^ in keeping with our results. Together, our findings suggest that monitoring BP changes, particularly SBP in women, could serve as a practical marker identifying concurrent cognitive decline.

Furthermore, sensitivity analyses suggest that the observed relationships between change in BP and changes in cognition are not readily explained by common medical, demographic, hormonal, or methodological factors. Adjustment for established medical (ie, BMI^34^ and *APOE ε4* status^39^, ^54^), sociodemographic (ie, race/ethnicity^37^), and other common metabolic, behavioral, and vascular factors, which differ by sex (eg, diabetes, high cholesterol, physical activity level, and smoking history, among others) known to influence the association between BP and cognition did not change the results. Although prior studies show BP declines before the onset of cognitive decline and dementia,^11^, ^15^ raising concerns of reverse causality, analyses excluding participants who developed dementia within 3 years of baseline yielded consistent results, suggesting reverse causality is a less likely explanation. Similarly, despite hypertension being the leading cause of mortality globally^55^ and sex differences in life expectancy,^42^ accounting for death did not change the results reasoning against survival bias as a primary explanation. Finally, although menopause at earlier ages is associated with increased risk for hypertension and earlier cognitive decline,^19^, ^43^, ^44^ adjusting for age at menopause did not change the results.

However, age‐stratified sensitivity analyses indicated that relationships between declining SBP and changes in cognition were selectively observed in those 70 years or older at baseline, suggesting the relationship is specific to late life aging. This corresponds to the age range when BP begins to decline,^6^, ^7^ and may reflect accumulated vascular damage, loss of compensatory cerebrovascular mechanisms, and among women, possible menopause‐related vascular changes (ie, loss of vasoprotective and neuroprotective effects related to estrogen^56^, ^57^, ^58^). Prior studies similarly support an age effect in the relationship between changes in BP and cognition. For example, 1 study showed no relationship between BP and cognition at follow‐up 11 years later in the youngest participants (<65 years old), while in those 75 years old or older higher BP was related to better cognition at follow‐up, with the strongest effect appearing in those over 85 years old.^46^ Overall, these findings indicate that the relationships between SBP decline and cognition may be concentrated in late life, when age‐related vascular vulnerability is greatest.

Building on these sensitivity analyses, several mechanisms not directly tested in this study may explain the observed sex differences in the relationship between SBP and cognition. Late‐life SBP decline may represent failing compensatory mechanisms needed to maintain cerebral perfusion and cognition. Chronic hypertension promotes arterial stiffening leading to impaired cerebral blood flow, contributing to cognitive decline.^59^, ^60^, ^61^ In this context, maintaining higher SBP may help preserve cerebral blood flow in the presence of arteriosclerosis‐induced resistance,^62^ whereas declining BP may reflect failure of these compensatory mechanisms. Regarding sex differences, hypertension is more strongly related in women to greater white matter hyperintensity burden, faster gray matter atrophy, and more severe white matter microstructural damage,^63^, ^64^, ^65^ suggesting BP disproportionately effects brain structure and cerebrovascular function in women. Menopause‐related hormonal changes may amplify this effect. During menopause the rapid decline in estradiol results in the loss of vasoprotective and neuroprotective effects,^56^, ^57^, ^58^, ^66^ accelerating vascular aging,^67^, ^68^ which may lead to increased susceptibility to the negative effects of declining SBP on the brain. In contrast, men experience more gradual hormonal declines and typically higher lifetime SBP, which may result in more prolonged vascular injury over time, reflected in our baseline associations between higher SBP and worse cognitive function but no relationships with declining SBP. Alternatively, lifelong disparities in hypertension detection, treatment, and management across the lifespan may contribute, as women are less likely to receive antihypertensive medication,^18^ seek urgent medical care,^69^ or discuss cardiovascular risk factors with their physicians.^69^ Differences in treatment approaches (eg, when to initiate medication, how aggressively to treat BP, how long to treat, etc.) may exist between women and men, possibly differentially impacting the relation of BP to cognition. These factors may contribute to poorer BP management in women over the lifespan, amplifying the impact of declining BP on cognition once the protective effects of estrogen are lost. Together, these potential biological and vascular aging differences, along with environmental/sociological differences, offer plausible explanations for why SBP decline in women is more strongly related to cognitive decline.

Finally, consistent with our hypothesis, cross‐sectional results showed that higher baseline levels of SBP and DBP were associated with worse global cognitive function in the full sample and in sex‐stratified models, although results were not uniform across cognitive domains. These findings consistent with a substantial body of literature indicating hypertension is associated with worse baseline cognitive function across various domains in older adults,^2^, ^37^ reinforcing the cross‐sectional association between higher levels of BP and lower levels of cognitive function in older adults.

This study had several limitations. Like many aging cohorts, our sample was predominantly White women, potentially limiting generalizability. Additionally, because our analyses examined simultaneous changes in BP and cognition, we are unable to determine the direction of the observed relationships (ie, whether declining BP precedes cognitive decline or vice‐versa) and, therefore, cannot determine whether declining BP represents a preclinical manifestation of neuropathology or contributes causally to cognitive decline. Additionally, although we assessed level and change in measured BP in our analyses, we did not capture the level of control of hypertension and future research would need to examine that aspect. Finally, because men had lower baseline cognitive scores compared to women, we cannot exclude that the observed sex differences are because of a floor effect of cognitive scores in men. Strengths of our study include the large, ongoing longitudinal cohorts with annual collection for over 31 years (1994–present), including annual BP measurements and a comprehensive battery of 19 neuropsychological tests across 5 cognitive domains. This study design enabled detailed assessment of cognitive and BP trajectories over multiple time points, allowing for more accurate and robust slope estimates than previous studies using only 2 time points. The use of a composite cognitive score minimized ceiling and floor effects compared to screening tools like the MMSE. Although prior studies were limited by small male sample sizes,^10^ our large male sample (n = 1,217) ensured sufficient statistical power. Additionally, extensive sensitivity analyses evaluated whether medical, demographic, and behavioral covariates or common sources of bias accounted for the observed associations, and results were robust across these analyses. Finally, joint mixed‐effects regression analyses enabled the examination of both levels and changes in BP and cognition simultaneously, leveraging multidimensional longitudinal data for more robust conclusions.

## Author Contributions

AWC, LLB, DAB, and ZA contributed to the conception and design of the study. NWB, AWC, and ZA contributed to the acquisition and analysis of data. NWB, AWC, and ZA contributed to drafting the text or preparing the figures.

## Potential Conflicts of Interest

Nothing to report.

## Supporting information


**Table S1.** Covariance parameter estimates of change in blood pressure with change in cognition and five cognitive domains by sex.

**Table S2.** Correlations of level and change of blood pressure with level and change of cognition and five cognitive domains by age group.

**Table S3.** Correlations of level and change of blood pressure with level and change of cognition and five cognitive domains by age group and sex.

## Data Availability

Raw data are available on request from qualified investigators applying through the RADC Research Resource Sharing Hub (www.radc.rush.edu/home.htm).
