# Supplementary material for: Sex Differences in the Association of Simultaneous Decline in Blood Pressure and Decline in Cognition during Aging
Source: Ann Neurol. 2026 Jul 7;100(3):572–83. doi: 10.1002/ana.78280 (PMC13495867; doi:10.1002/ana.78280)
Supplement: Supplementary file 1 [file ANA-100-572-s001.docx]

| **Table S2. Correlations of Level and Change of Blood Pressure with Level and Change of Cognition and Five Cognitive Domains by Age Group** | | | | | | | | | |
| --- | --- | --- | --- | --- | --- | --- | --- | --- | --- |
|  |  | **≤70 years old at baseline**  **(n = 1063)** | | | | **>70 years old at baseline**  **(n = 3656)** | | | |
|  | | **Level, Level** | | **Change, Change** | | **Level, Level** | | **Change, Change** | |
|  |  | **r value** | **p-value** | **r value** | **p-value** | **r value** | **p-value** | **r value** | **p-value** |
| Systolic Pressure | Global Cognition | -0.05 | 0.22 | 0.11 | 0.10 | -0.04 | 0.06 | **0.22**** | **< 0.001** |
|  | Working Memory | 0.03 | 0.39 | 0.11 | 0.12 | -0.03 | 0.10 | **0.26**** | **< 0.001** |
|  | Semantic Memory | -0.02 | 0.69 | 0.07 | 0.31 | **-0.05*** | **0.03** | **0.23**** | **< 0.001** |
|  | Episodic memory | -0.06 | 0.11 | 0.08 | 0.21 | -0.01 | 0.75 | **0.16**** | **< 0.001** |
|  | Perceptual Speed | -0.06 | 0.12 | 0.12 | 0.10 | **-0.07**** | **< 0.001** | **0.18**** | **< 0.001** |
|  | Visuospatial Ability | -0.06 | 0.12 | 0.08 | 0.27 | **-0.06**** | **0.005** | **0.18**** | **< 0.001** |
| Diastolic Pressure | Global Cognition | -0.07 | 0.06 | -0.03 | 0.70 | **-0.06**** | **0.004** | -0.04 | 0.38 |
|  | Working Memory | -0.01 | 0.86 | -0.04 | 0.59 | **-0.06**** | **0.004** | -0.04 | 0.38 |
|  | Semantic Memory | -0.05 | 0.21 | -0.06 | 0.44 | -0.04 | 0.09 | -0.02 | 0.62 |
|  | Episodic memory | **-0.08*** | **0.03** | -0.05 | 0.43 | -0.04 | 0.06 | -0.05 | 0.23 |
|  | Perceptual Speed | -0.07 | 0.06 | 0.00 | 0.96 | **-0.05*** | **0.02** | 0.01 | 0.88 |
|  | Visuospatial Ability | -0.04 | 0.31 | -0.01 | 0.91 | **-0.06**** | **0.003** | 0.00 | 0.98 |
| Results of age-stratified bivariate mixed effects models jointly modeling both change in blood pressure and cognition over time. P-value reflects the covariance parameter estimate (data not shown). Significant correlations are **bolded** for clarity (* = p <0.05, ****** = p <0.01). | | | | | | | | | |
