# Supplementary material for: Sex Differences in the Association of Simultaneous Decline in Blood Pressure and Decline in Cognition during Aging
Source: Ann Neurol. 2026 Jul 7;100(3):572–83. doi: 10.1002/ana.78280 (PMC13495867; doi:10.1002/ana.78280)
Supplement: Supplementary file 2 [file ANA-100-572-s002.docx]

| **Table S3. Correlations of Level and Change of Blood Pressure with Level and Change of Cognition and Five Cognitive Domains by Age Group and Sex** | | | | | | | | | | | | | | | | | | | |
| --- | --- | --- | --- | --- | --- | --- | --- | --- | --- | --- | --- | --- | --- | --- | --- | --- | --- | --- | --- |
|  | | **≤70 years old at baseline (n = 1063)** | | | | | | | | **>70 years old at baseline (n = 3656)** | | | | | | | | |  |
|  |  | **Women (n = 798)** | | | | **Men (n = 265)** | | | | **Women (n = 2704)** | | | | **Men (n = 952)** | | | |  |  |
|  |  | **Level, Level** | | **Change, Change** | | **Level, Level** | | **Change, Change** | | **Level, Level** | | **Change, Change** | | **Level, Level** | | **Change, Change** | |  |  |
|  |  | **r value** | **p-value** | **r value** | **p-value** | **r value** | **p-value** | **r value** | **p-value** | **r value** | **p-value** | **r value** | **p-value** | **r value** | **p-value** | **r value** | **p-value** |  |  |
| Systolic Pressure | Global Cognition | -0.06 | 0.17 | 0.11 | 0.13 | -0.03 | 0.71 | 0.04 | 0.76 | -0.03 | 0.30 | **0.29†** | **< 0.001** | **-0.09*** | **0.03** | -0.02 | 0.79 |  |  |
|  | Working Memory | 0.01 | 0.90 | **0.18*** | **0.04** | 0.10 | 0.20 | -0.09 | 0.50 | -0.02 | 0.44 | **0.34†** | **< 0.001** | **-0.09*** | **0.03** | -0.01 | 0.92 |  |  |
|  | Semantic Memory | -0.04 | 0.40 | 0.04 | 0.64 | 0.03 | 0.66 | 0.15 | 0.25 | -0.05 | 0.06 | **0.29†** | **< 0.001** | -0.05 | 0.20 | 0.05 | 0.61 |  |  |
|  | Episodic memory | -0.06 | 0.20 | 0.05 | 0.49 | -0.10 | 0.21 | 0.15 | 0.24 | 0.00 | 0.92 | **0.23†** | **< 0.001** | -0.04 | 0.28 | -0.08 | 0.36 |  |  |
|  | Perceptual Speed | -0.07 | 0.12 | 0.15 | 0.06 | -0.03 | 0.72 | 0.01 | 0.93 | **-0.07*** | **0.01** | **0.23†** | **< 0.001** | **-0.08*** | **0.04** | 0.06 | 0.47 |  |  |
|  | Visuospatial Ability | -0.09 | 0.06 | **0.22*** | **0.02** | 0.00 | 0.98 | -0.24 | 0.09 | **-0.05*** | **0.05** | **0.21†** | **< 0.001** | **-0.09*** | **0.03** | 0.06 | 0.50 |  |  |
| Diastolic Pressure | Global Cognition | **-0.09*** | **0.04** | -0.06 | 0.45 | -0.01 | 0.86 | 0.08 | 0.54 | -0.04 | 0.09 | 0.00 | 0.94 | **-0.13†** | **0.002** | **-0.18*** | **0.03** |  |  |
|  | Working Memory | -0.02 | 0.63 | -0.02 | 0.83 | 0.03 | 0.66 | -0.12 | 0.42 | **-0.05*** | **0.04** | 0.01 | 0.91 | **-0.09*** | **0.02** | **-0.19*** | **0.03** |  |  |
|  | Semantic Memory | -0.08 | 0.07 | -0.11 | 0.21 | 0.06 | 0.42 | 0.10 | 0.47 | -0.02 | 0.40 | 0.01 | 0.77 | **-0.08*** | **0.05** | -0.15 | 0.09 |  |  |
|  | Episodic memory | **-0.09*** | **0.05** | -0.12 | 0.13 | -0.07 | 0.36 | 0.17 | 0.19 | -0.02 | 0.40 | -0.02 | 0.70 | **-0.11*** | **0.01** | -0.16 | 0.05 |  |  |
|  | Perceptual Speed | -0.06 | 0.16 | 0.00 | 0.99 | -0.10 | 0.23 | -0.04 | 0.88 | -0.03 | 0.21 | 0.03 | 0.49 | **-0.10*** | **0.01** | -0.06 | 0.43 |  |  |
|  | Visuospatial Ability | **-0.09*** | **0.05** | -0.01 | 0.92 | 0.11 | 0.16 | -0.02 | 0.88 | -0.05 | 0.06 | 0.00 | 0.97 | **-0.11*** | **0.01** | 0.00 | 0.98 |  |  |
| Results of bivariate mixed effects models jointly modeling both change in blood pressure and cognition over time, stratified by both age and sex. P-value reflects the covariance parameter estimate (data not shown). Significant correlations are **bolded** for clarity (* = p <0.05, ****** = p <0.01). | | | | | | | | | | | | | | | | | | | |
