# Supplementary material for: Sex Differences in the Association of Simultaneous Decline in Blood Pressure and Decline in Cognition during Aging
Source: Ann Neurol. 2026 Jul 7;100(3):572–83. doi: 10.1002/ana.78280 (PMC13495867; doi:10.1002/ana.78280)
Supplement: Supplementary file 3 [file ANA-100-572-s003.docx]

| **Table S1. Covariance Parameter Estimates of Change in Blood Pressure with Change in Cognition and Five Cognitive Domains by Sex** | | | | | | | | | | | | |
| --- | --- | --- | --- | --- | --- | --- | --- | --- | --- | --- | --- | --- |
|  |  | **Women** | | | | | **Men** | | | | |  |
| **Bivariate Outcomes** | | **Estimate (± SE)** | **Z Value** | **Lower** | **Upper** | **Estimate (± SE)** | | **Z Value** | **Lower** | **Upper** |  |  |
| Systolic Pressure | Global Cognition | **0.00133 ± 0.00021** | **6.22**** | **0.00091** | **0.00175** | 0.00003 ± 0.00037 | | 0.09 | -0.00069 | 0.00075 |  |  |
|  | Working Memory | **0.00109 ± 0.00016** | **6.88**** | **0.00078** | **0.00140** | -0.00010 ± 0.00025 | | -0.40 | -0.00058 | 0.00039 |  |  |
|  | Semantic Memory | **0.00157 ± 0.00028** | **5.60**** | **0.00102** | **0.00212** | 0.00061 ± 0.00050 | | 1.21 | -0.00038 | 0.00159 |  |  |
|  | Episodic memory | **0.00119 ± 0.00024** | **4.89**** | **0.00071** | **0.00167** | -0.00012 ± 0.00040 | | -0.30 | -0.00090 | 0.00066 |  |  |
|  | Perceptual Speed | **0.00086 ± 0.00017** | **4.96**** | **0.00052** | **0.00120** | 0.00019 ± 0.00027 | | 0.71 | -0.00033 | 0.00071 |  |  |
|  | Visuospatial Ability | **0.00071 ± 0.00016** | **4.48**** | **0.00040** | **0.00103** | -0.00005 ± 0.00025 | | -0.22 | -0.00054 | 0.00043 |  |  |
| Diastolic Pressure | Global Cognition | -0.00004 ± 0.00020 | -0.20 | -0.00044 | 0.00036 | -0.00063 ± 0.00038 | | -1.66 | -0.00137 | 0.00011 |  |  |
|  | Working Memory | 0.00000 ± 0.00015 | 0.01 | -0.00029 | 0.00029 | **-0.00058 ± 0.00026** | | **-2.24*** | **-0.00109** | **-0.00007** |  |  |
|  | Semantic Memory | -0.00008 ± 0.00027 | -0.29 | -0.00060 | 0.00045 | -0.00053 ± 0.00052 | | -1.04 | -0.00154 | 0.00047 |  |  |
|  | Episodic memory | -0.00022 ± 0.00023 | -0.94 | -0.00067 | 0.00024 | -0.00055 ± 0.00041 | | -1.35 | -0.00136 | 0.00025 |  |  |
|  | Perceptual Speed | 0.00012 ± 0.00017 | 0.75 | -0.00020 | 0.00045 | -0.00016 ± 0.00028 | | -0.58 | -0.00070 | 0.00038 |  |  |
|  | Visuospatial Ability | 0.00004 ± 0.00015 | 0.28 | -0.00025 | 0.00034 | -0.00001 ± 0.00026 | | -0.04 | -0.00052 | 0.00050 |  |  |
| Results of bivariate mixed-effects models jointly modeling both change in blood pressure and cognition over time, adjusted for age, education, and antihypertensive medication use. Lower and upper bounds of 95%CI from bivariate mixed-effects models are presented for each variable. Significant results are **bolded** for clarity. (* = p <0.05, ****** = p <0.01). | | | | | | | | | | | | |
